# Eyes of Understanding: Ophthalmologists' Attitudes Towards Caring for People With Intellectual Disabilities in Clinical and Nonclinical Settings

**DOI:** 10.1111/jar.70128

**Published:** 2025-09-25

**Authors:** Chiun‐Ho Hou, Yueh‐Ching Chou, Christy Pu

**Affiliations:** ^1^ Department of Ophthalmology National Taiwan University Hospital Taiwan; ^2^ Institute of Public Health, School of Medicine National Yang Ming Chiao Tung University Taipei Taiwan; ^3^ Department of Social Work Asia University Taichung Taiwan

**Keywords:** attitudes, disparity, eye care, intellectual disability, ophthalmologists, vision health

## Abstract

**Background:**

This study compared ophthalmologists' and other health‐care professionals' attitudes towards people with intellectual disabilities in clinical and nonclinical settings.

**Methods:**

Between January 2022 and October 2023, a survey was conducted among 127 physicians, 100 optometrists/opticians, and 86 other health‐care professionals in Taiwan. Their attitudes towards people with intellectual disabilities in nonclinical and clinical settings were examined using 14‐item and 8‐item scales, respectively; data on their training and care experience were collected.

**Results:**

Ophthalmologists exhibited positive attitudes in nonclinical settings (mean = 55.9/70) but exhibited less positive attitudes in clinical settings (mean = 23.5/40). In clinical settings, ophthalmologists exhibited the highest scores across most items, indicating the most favourable attitudes. Optometrists generally had the second‐highest scores, followed by other healthcare professionals.

**Conclusion:**

Ophthalmologists' attitudes towards people with intellectual disabilities may considerably differ between clinical and nonclinical settings.

**Summary**

- Ophthalmologists in this study have more negative attitudes towards people with intellectual disabilities in clinical settings than they do in non‐clinical settings.
- Ophthalmologists in this study have more positive attitudes towards people with intellectual disabilities than optometrists do.
- Specialised training alone does not improve the attitude of ophthalmologists in this study and other health professionals towards people with intellectual disabilities in clinical settings.

## 1 Introduction

People with intellectual disabilities are at high risk of ocular conditions (Serra et al. 2020). Vision problems are more prevalent among these people than among those without intellectual disabilities (Owens et al. 2006). Avoidable visual impairment is common among people with intellectual disabilities (Emerson and Robertson 2011). Donaldson et al. (2019) reported that 44% of pupils in English special schools had no previous eye‐care history despite a substantial need for refractive correction. People with intellectual disabilities also show high rates of refractive error, strabismus, and lens opacities (van Splunder et al. 2004). Vision problems can limit social and cognitive development in people with intellectual disabilities (Chokron et al. 2020). Accordingly, adequate and appropriate vision care for people with intellectual disabilities is essential to ensure equity in the provision of vision health care.

A review article revealed significant disparities in access to and the quality of general health care received between the general population and people with intellectual disabilities; the review also reported that regarding eye care, people with intellectual disabilities often face challenges in receiving the same standard of care as the general population when seeking medical treatment (Ellison 2013). Moreover, a study reported that people with intellectual disabilities are less likely to receive preventive and early treatment for eye conditions (Owens et al. 2006). However, research examining whether disparities in eye care for people with intellectual disabilities are related to the attitudes of ophthalmologists and other health professionals towards caring for these people is limited.

### 1.1 Physicians' Attitudes Towards People With Intellectual Disabilities

The public often exhibits negative attitudes towards people with intellectual disabilities (Scior 2011). Given this context, physicians' attitudes towards people with intellectual disabilities are crucial because they can contribute to the aforementioned health‐care disparities (Ditchman et al. 2013). Physicians' attitudes towards people with intellectual disabilities can considerably affect the quality and accessibility of health‐care services offered to these people (Bacherini et al. 2021). A systematic review revealed that physicians generally possess moderate to good knowledge about intellectual disabilities and related conditions; however, their knowledge is incomplete regarding the rights and abilities of people with intellectual disabilities and tailoring health‐care practices to this population (Bacherini et al. 2021). In addition, Kerins et al. (2004) reported that the majority of physicians believe that caring for people with intellectual disabilities is more difficult than caring for those people without intellectual disabilities. Physicians tend to avoid treating people with intellectual disabilities (Bacherini et al. 2021). Physicians' proficiency in caring for people with intellectual disabilities can be affected by the physicians lacking adequate training and education on these people, challenges with communication, and disruptions in the continuity of health care (Kerins et al. 2004).

### 1.2 Ophthalmologists and Other Health‐Care Professionals in Eye Care

Eye care is provided by multiple professionals, including ophthalmologists, optometrists, opticians, and allied health‐care professionals (Stevens et al. 2000). Whether ophthalmologists' attitudes towards people with intellectual disabilities differ from those of optometrists and other health‐care professionals should be investigated; this is because in Taiwan, ophthalmologists are often the first point of contact for people with vision problems and play major roles in treatment decisions for these people. In addition, during their medical education, ophthalmologists receive comprehensive training in whole‐body medicine and psychiatry; thus, they possess more knowledge about intellectual disabilities or systemic issues compared with other health‐care professionals. Accordingly, ophthalmologists' knowledge about intellectual disabilities may contribute to fostering positive attitudes towards people with intellectual disabilities.

### 1.3 Gaps in the Literature

The literature on the provision of care for people with intellectual disabilities contains major gaps that warrant attention. First, although numerous studies have documented that physicians and health‐care personnel may exhibit negative attitudes towards people with disabilities, particularly those with intellectual disabilities (Kerins et al. 2004; Lagu et al. 2022), few have investigated this issue in the ophthalmic care setting. Because vision is strongly related to quality of life, whether ophthalmologists have positive attitudes towards people with intellectual disabilities must be determined.

Second, studies have yet to compare physicians' attitudes towards people with intellectual disabilities in clinical settings with their attitudes towards these people in nonclinical settings. Such a comparison is crucial because physicians can hold generally positive attitudes towards people with intellectual disabilities—for example, they might advocate for their integration into the community—but they may not accommodate the specific needs of people with intellectual disabilities in clinical settings. A potential reason for this discrepancy is the lack of financial incentives owing to the reimbursement system in health insurance programmes (Portney et al. 2023). Therefore, in terms of providing care, ophthalmologists might exhibit negative attitudes towards people with intellectual disabilities in clinical settings compared with the attitudes in nonclinical settings. This difference may be attributed to the direct responsibilities of ophthalmologists in providing clinical care for people with vision problems and the potential impact on their income.

Physicians are also expected to make treatment decisions that focus on symptom reduction or problem resolution (Bowling et al. 2012). From a methodological perspective, studies on physicians' attitudes towards people with intellectual disabilities have rarely included comparison groups; thus, determining whether physicians exhibit positive or negative attitudes towards people with intellectual disabilities compared with other health‐care professionals is difficult. Similarly, ophthalmologists have distinct roles in providing eye care for people with intellectual disabilities compared with other health‐care professionals. However, no study has examined whether their attitudes are positive or negative relative to those of other health‐care professionals; thus, this aspect remains largely unexplored.

### 1.4 Theoretical Framework

Social cognitive theory (SCT) (Bandura 1986) provides a framework for understanding how and why physicians' attitudes towards people with intellectual disabilities differ between clinical and nonclinical settings. SCT emphasises that human behaviour is influenced by the interactions of personal factors, environmental factors, and behavioural factors, and this framework can be applied to explain why physicians exhibit contrasting attitudes depending on the context. In nonclinical settings, physicians may exhibit positive attitudes towards people with intellectual disabilities, and this is driven by social norms, altruistic values, and their professional identity. However, in clinical settings, practical constraints such as financial disincentives, time limitations, and a lack of resources may hinder their willingness to treat people with intellectual disabilities. Physicians may also perceive a higher level of clinical risk when managing persons with intellectual disabilities, due to challenges in obtaining reliable assessments such as full visual fields or accurate visual acuities.

The social inclusion concept can also be used to explain physicians' attitudes towards people with intellectual disabilities. Social inclusion emphasises the importance of integrating marginalised individuals into society's mainstream and providing equal opportunities for these individuals (Hall 2010). Factors influencing social inclusion include physical and social independence, social interaction, cultural attitudes, and functional independence (Merrells et al. 2018). To improve care for people with intellectual disabilities, physicians should undergo experiential learning and be exposed to these people (Wilkinson et al. 2012). Enhancing physician education and promoting positive community attitudes may lead to more satisfactory health‐care experiences and increased social participation for people with intellectual disabilities (Kerins et al. 2004; Merrells et al. 2018).

### 1.5 Research Question and Hypotheses

To address these gaps, we conducted a study in Taiwan comparing ophthalmologists' attitudes towards people with intellectual disabilities in clinical settings with their attitudes in non‐clinical settings. We further compared the ophthalmologists' scores with those of other health‐care professionals.

Our research questions are outlined as follows: (1) Do ophthalmologists exhibit different attitudes towards people with intellectual disabilities in clinical versus nonclinical settings? (2) Do ophthalmologists' attitudes towards people with intellectual disabilities differ from those of other health‐care professionals? (3) Is having experience in caring for people with intellectual disabilities, having family members or friends with intellectual disabilities, or having participated in activities or workshops planned by organisations for people with intellectual disabilities associated with more positive attitudes towards people with intellectual disabilities?

On the basis of our literature review, we proposed the following hypotheses:

*Ophthalmologists exhibit negative attitudes towards people with intellectual disabilities in clinical settings compared with the attitudes in nonclinical settings*.

*Ophthalmologists exhibit positive attitudes towards people with intellectual disabilities compared with other health‐care professionals*.

*Having experience in caring for people with intellectual disabilities, having family members or friends with intellectual disabilities, or having participated in activities or workshops planned by organisations for people with intellectual disabilities is associated with more positive attitudes towards people with intellectual disabilities*.

## 2 Methods

### 2.1 Participants

We employed convenience sampling for participant selection. Members of the Ophthalmological Society of Taiwan and the Taiwan Optometry Education Association were invited to participate in our questionnaire survey. We also distributed the questionnaire during several general meetings and specific meetings held by the aforementioned organisations and to hospitals and governmental organisations. When distributed onsite at general meetings and specific meetings, the survey was self‐administered in paper form or through a QR code that linked to the survey. This study recruited ophthalmologists, occupational therapists, optometrists/opticians, special education teachers, orientation and mobility trainers, and social workers who provide care for people with intellectual disabilities. Although non–health‐care professionals attending professional conferences could access the survey, their data were excluded from the analysis; specifically, our analysis included only the data of health‐care professionals, who were identified from their self‐reported occupation in the questionnaire. For questionnaires distributed to hospitals and governmental organisations, participants were invited through email.

In Taiwan, the number of ophthalmologists nationwide is 1790, and the number of ophthalmologists who participated in this study was 127 (7.09%). Moreover, the number of optometrists/opticians nationwide is 13,179, and the number of optometrists/opticians who participated in this study was 100 (0.76%). This study included a total of 313 participants. Specifically, 50 participants were recruited at a meeting pertaining to cataract and refractive surgery, 75 were recruited at a myopia symposium, and 96 were recruited from training programmes for medical personnel providing eye care for people with intellectual disabilities. Moreover, 92 were invited by the authors through email.

### 2.2 Definition of Intellectual Disabilities in Taiwan

In Taiwan, to receive disability welfare benefits, people with intellectual disabilities must complete an official registration process for obtaining their medical diagnoses. In this process, medical professionals evaluate IQ scores, neurological examination outcomes, mental health status, self‐care abilities, communication skills, and maladaptive behaviours. On the basis of these assessments, intellectual disabilities are categorised into four levels: profound, severe, moderate, and mild (Taipei City Government 2024).

### 2.3 Ethical Considerations

Before taking the survey, potential participants provided informed consent using either a paper form or an online form, depending on the mode of data collection. People who refused to provide consent did not receive the survey. Participants did not receive any compensation, and they had no relationship with the authors of this study that could influence the study findings. Data were stored on a secure computer protected by passwords, and only members approved by the Institutional Review Board (IRB) could have access to the data.

### 2.4 Survey Instrument

The designed questionnaire comprised four sections, which are described as follows.

#### 2.4.1 General Attitude Towards People With Intellectual Disabilities

This section was used to evaluate the respondents' general attitudes towards people with intellectual disabilities in nonclinical settings. The items were adapted from a scale that was used by Tachibana and Watanabe (2003) to study attitudes towards people with intellectual disabilities. The original scale had 18 items. We selected only the first 14 items because the other 4 items were not considered to be relevant to the local context. The response to each item was provided on a 5‐point rating scale, with 1 representing ‘strongly disagree’ and 5 representing ‘strongly agree’. The total score for this section was 70 points. The 14 items are presented in Appendix A. In the current study, the Cronbach's α for these 14 items was 0.824 (*n* = 313).

#### 2.4.2 Clinical Setting Attitude

This section was designed to present a likely situation in a clinical setting involving people with intellectual disabilities as well as with visual impairment. Because no scale has been created that would be appropriate for the context of this study, our research team developed one. The items were formulated by an expert panel comprising 10 members, with the members including ophthalmologists, optometrists, front‐line health‐care workers supporting people with intellectual disabilities, and academic scholars including the researcher in intellectual disability studies. A total of eight items were devised to evaluate participants' attitudes and anticipated responses in a clinical setting for a hypothetical service user with intellectual disabilities named Chang. This service user is described as follows:

Chang, who is 30 years old, has been given a diagnosis of severe intellectual disability combined with physical disability, which makes it difficult for him to walk for a long time. He cannot concentrate, and his eyes cannot focus. He also has abnormal refractive errors, exotropia, and pale optic nerves. He cannot communicate with others through language and cannot comply with medical instructions for visual examinations. However, he can understand colours. He cannot sit still for more than 3 min and easily feels scared in unfamiliar environments and around strangers; therefore, he is used to carrying a small booklet with him. He may use his limbs to touch strangers he is interested in to express curiosity. Regarding medical care, he feels scared when he sees unfamiliar medical staff and equipment in the consultation room, and he is unwilling to cooperate with treatment or wear glasses.

The items were methodically structured with an ascending order of positivity in their phrasing; the first item presented a negative assertion, the second introduced a neutral statement, and the subsequent items incrementally conveyed more favourable attitudes. The eight items, in order, are as follows: ‘I would wish Chang were not my patient’; ‘To me, there would be no difference between Chang and my other patients’; ‘I would be happy to have Chang as my patient, even if treating him took more time than treating other patients would’; ‘I would take pride in having Chang as my patient’; ‘Patients like Chang require special expertise, so I would refer them to other professionals’; ‘Patients like Chang require special expertise, so I would seek to enhance my knowledge in this domain’; ‘Although more time would be required to do so, I would feel a sense of accomplishment in treating unique patients like Chang’; and ‘I would like to conduct further research on unique patients like Chang’. Each of these items is rated on a 5‐point scale, with 1 indicating ‘strongly disagree’ and 5 indicating ‘strongly agree’. The total score for this section was 40 points. In the current study, the Cronbach's *α* was 0.913. The Cronbach's *α* value was calculated on the basis of the responses to the survey (*n* = 313).

#### 2.4.3 Experience in Taking Care of People With Intellectual Disabilities

We presented three experience‐related questions to the participants to ascertain their experience with people with intellectual disabilities. These three questions are as follows: ‘Have you had experience in treating or taking care of a patient with intellectual disabilities?’; ‘Do you have any family members or friends with intellectual disabilities?’; and ‘Have you ever participated in activities or workshops organized by organizations for people with intellectual disabilities?’ For all three questions, the answers were dichotomised as ‘Yes’ or ‘No’.

### 2.5 Statistical Analysis

We calculated descriptive statistics for the scores derived for attitudes towards people with intellectual disabilities in clinical and nonclinical settings. For both settings, a higher score indicated more favourable attitudes towards people with intellectual disabilities. We divided the participants into three groups: ophthalmologists, optometrist/opticians, and others. Subsequently, we stratified them according to their responses to the three experience‐related questions. Linear regression analyses were then conducted. In the regression analyses, the dependent variables were the total score for attitudes in clinical settings and that for attitudes in nonclinical settings; the independent variables were age, age squared (to account for any nonlinear effect of age), sex, religion (Dao, Buddhist, Christian, or other), educational attainment (vocational schools, college or graduate school), marital status (single, married, or other), place of practice (medical centre, regional hospital, district hospital, or clinic), prior experience in caring for people with intellectual disabilities (yes or no), years of practice and its squared value, and professional designation (ophthalmologist, optometrist/optician, or other). Finally, we used the linear regression model to estimate predicted scores. For estimating the scores, we substituted the values of the independent variables into the regression equation and then multiplied each variable by its corresponding coefficient; the results were summed, and the intercept was added to obtain the predicted value. All statistical analyses were conducted using STATA 15 (StataCorp 2017).

## 3 Results

Table 1 presents the participants' characteristics. A total of 127 ophthalmologists, 100 optometrists/opticians, and 86 other health‐care professionals responded to the survey. We observed significant differences in the sociodemographic characteristics between the three groups. The ophthalmologist group was significantly older, had a lower proportion of men, and had a lower proportion of single individuals compared with the optometrist/optician and other health‐care professional groups. No marked differences in religious affiliations emerged between the groups. Because of their educational trajectory, most of the optometrists/opticians had vocational schools as their highest educational attainment. Among the ophthalmologists in our sample, 49.6% practised in medical centres.

**TABLE 1:** Sample characteristics.

	Ophthalmologist		Optometrist/Optician		Others		*p* ^a^
	*n* = 127	%	*n* = 100	%	*n* = 86	%	
Age (mean/SD)	41.77	10.9	35.13	10.9	38.08	10.7	< 0.001
Sex (male)	51	40.2	52	52.0	72	83.7	< 0.001
Marital status							
Single	13	10.2	54	54.0	52	60.5	< 0.001
Married	53	41.7	40	40.0	32	37.2	
Others	61	48.0	6	6.0	2	2.3	
Religion							
None	61	48.0	44	44.0	35	40.7	0.609
Dao	39	30.7	33	33.0	36	41.9	
Buddhist	16	12.6	15	15.0	7	8.1	
Christian/Catholic	11	8.7	8	8.0	8	9.3	
Education							
Vocational school	0	0.0	70	70.0	63	73.3	
College	95	74.8	30	30.0	21	24.4	< 0.001
Graduates	32	25.2	0	0.0	2	2.3	
Place of practice							
Medical centre	63	49.6	4	4.0	20	23.3	< 0.001
Regional hospitals	28	22.1	2	2.0	35	40.7	
District hospitals	5	3.9	3	3.0	5	5.8	
Clinics	31	24.4	15	15.0	7	8.1	
Others	0	0.0	76	76.0	19	22.1	
Years of practice	12.52	10.3	9.40	9.4	13.02	10.9	0.0268

^a^ *p*‐value derived using ANOVA for continuous variables and chi‐squared test for categorical variables.

Table 2 presents the scores for the participants' attitudes towards people with intellectual disabilities in nonclinical settings. The scores were recoded such that a higher score indicated a more positive attitude. A 5‐point Likert scale was used, with a score of three indicating a neutral answer. The results reveal that only the responses for the item ‘People who with intellectual disabilities pose a threat to society because they can pass on their genes for low intelligence’ differed between the three groups (*p* < 0.001). The ophthalmologists had the highest average score (mean = 4.09; SD = 0.97). Optometrist/optician had an average score of 3.53 (SD = 1.24). Other health‐care professionals had the lowest average score (mean = 3.48; SD = 1.12). Overall, the average scores for all three occupational groups were higher than three, indicating that the participants in all three occupational groups exhibited positive attitudes towards people with intellectual disabilities.

**TABLE 2:** General attitude scores.

	Ophthalmologist		Optometrist/Optician		Other		*p*
	*n* = 127		*n* = 100		*n* = 86		
	Mean	SD	Mean	SD	Mean	SD	
1. The best care for individuals with intellectual disability is inclusion in normal life activities in a community	4.33	0.75	4.40	0.75	4.52	0.59	0.152
2. Individuals with intellectual disability pose a threat to society because they can pass on their genes for low intelligence	4.09	0.97	3.53	1.24	3.48	1.12	< 0.001
3. Individuals with intellectual disability can have close personal relationships, just like everyone else	4.34	0.80	4.29	0.90	4.42	0.79	0.572
4. I would not consider dating someone who has a family member with an intellectual disability	3.84	1.04	3.96	1.13	3.88	1.07	0.715
5. Public awareness of the reality of the lives of individuals with intellectual disability should be increased	4.28	0.86	4.53	0.76	4.44	0.81	0.058
6. Facilities for individuals with intellectual disability should be kept out of residential areas	4.34	0.82	4.25	0.89	4.41	0.87	0.456
7. I do not mind having a tax increase if the money will be used for social services for individuals with intellectual disability	3.46	0.90	3.44	1.02	3.49	1.09	0.946
8. I would not want to live next door to individuals with intellectual disability who live in the same apartment building	4.04	0.85	4.06	1.01	4.01	1.01	0.942
9. I am willing to allow my child to be close personal friends with children with intellectual disability	4.02	0.95	4.15	0.88	4.23	0.75	0.216
10. I would rather not work with individuals with intellectual disability	3.80	1.00	3.97	1.11	4.12	0.86	0.079
11. Although recent TV programs and newspapers have included topics concerning individuals with intellectual disability, I am not interested because I have no relationship with such people	4.00	0.85	4.07	1.03	4.14	1.01	0.575
12. I would rather my child not sit next to a child with intellectual disability in a school classroom because they may disturb my child's learning	4.04	0.89	4.11	0.97	4.14	0.91	0.712
13. Individuals without intellectual disability should meet more frequently with individuals with intellectual disability	3.57	0.85	3.66	0.95	3.67	0.89	0.666
14. I wish to avoid getting involved with individuals with intellectual disability, although I have sympathy for them and their families	3.86	0.96	3.93	1.08	4.00	0.91	0.584

Table 3 presents the scores for the participants' attitudes towards a hypothetical service user with intellectual disabilities in a clinical setting. The scores were recoded such that a higher score indicated a more positive attitude. The results reveal significant differences in the scores for all items between the three groups. For the item ‘I would wish Chang were not my patient’, the ophthalmologists had an average score of 3.05 (SD = 1.13), whereas the optometrist/opticians had an average score of only 2.26 (SD = 1.23). For the items ‘To me, there would be no difference between Chang and my other patients’, ‘I would be happy to have Chang as my patient, even if treating him took more time than treating other patients would’, ‘I would take pride in having Chang as my patient’, ‘Patients like Chang require special expertise, so I would refer them to other professionals’, and ‘I would like to conduct further research on unique patients like Chang’, the average score for the ophthalmologists was lower than three. Among the three groups, the ophthalmologists had the highest mean scores for all items, with the exception of the item ‘Patients like Chang require special expertise, so I would refer them to other professionals’.

**TABLE 3:** Clinical setting score by occupation.^a^

	Ophthalmologist		Optometrist/optician		Other		*p* ^b^
	*n* = 127		*n* = 100		*n* = 86		
	Mean	SD	Mean	SD	Mean	SD	
1. I would wish Chang were not my patient	3.05	1.13	2.26	1.23	1.95	0.97	< 0.001
2. To me, there would be no difference between Chang and my other patients	2.94	1.20	2.27	1.25	2.24	1.21	< 0.001
3. I would be happy to have Chang as my patient, even if treating him took more time than treating other patients would	2.99	0.97	2.28	1.23	2.01	0.89	< 0.001
4. I would take pride in having Chang as my patient	2.98	0.80	2.43	1.16	2.28	0.93	< 0.001
5. Patients like Chang require special expertise, so I would refer them to other professionals	3.42	1.20	2.38	1.29	2.24	1.25	< 0.001
6. Patients like Chang require special expertise, so I would seek to enhance my knowledge in this domain	3.29	1.13	2.14	1.41	1.58	0.76	< 0.001
7. Although more time would be required to do so, I would feel a sense of accomplishment in treating unique patients like Chang	3.12	1.02	2.18	1.37	1.80	0.88	< 0.001
8. I would like to conduct further research on unique patients like Chang	2.97	1.00	2.21	1.31	1.90	0.89	< 0.001

^a^ Scores were reversed such that a higher score indicated a more positive attitude towards individuals with intellectual disability.

^b^ Comparison of means among the three groups through ANOVA.

Table 4 presents the results obtained after the participants were stratified according to their responses to the three experience‐related items. Among the participants, 48.9% reported having experience in care for people with intellectual disabilities, 26.8% reported having family members or friends with intellectual disabilities, and 27.5% % reported having previously participated in activities or workshops organised by organisations for people with intellectual disabilities.

**TABLE 4:** Scenario score by experience.^a^

	Had experienced caring for patients with ID^b^					Had family or friends with ID^c^					Had training experience^d^				
	No *n* = 160		Yes *n* = 153		*p*	No *n* = 229		Yes *n* = 84		*p*	No *n* = 227		Yes *n* = 86		*p*
	Mean	SD	Mean	SD		Mean	SD	Mean	SD		Mean	SD	Mean	SD	
1. I would wish Chang were not my patient	2.65	1.18	2.33	1.23	0.021	2.52	1.21	2.43	1.23	0.558	2.64	1.17	2.10	1.26	< 0.001
2. To me, there would be no difference between Chang and my other patients	2.64	1.26	2.42	1.25	0.124	2.55	1.27	2.49	1.23	0.679	2.64	1.23	2.27	1.31	0.020
3. I would be happy to have Chang as my patient, even if treating him took more time than treating other patients would	2.61	1.11	2.37	1.13	0.060	2.49	1.11	2.50	1.19	0.964	2.67	1.09	2.03	1.11	< 0.001
4. I would take pride in having Chang as my patient	2.66	1.04	2.57	0.97	0.442	2.60	1.00	2.65	1.02	0.661	2.70	0.97	2.37	1.06	0.009
5. Patients like Chang require special expertise, so I would refer them to other professionals	3.21	1.35	3.26	1.37	0.750	3.25	1.34	3.20	1.40	0.789	3.15	1.32	3.47	1.44	0.066
6. Patients like Chang require special expertise, so I would seek to enhance my knowledge in this domain	2.56	1.38	2.34	1.32	0.146	2.44	1.34	2.50	1.40	0.714	2.62	1.32	2.02	1.35	0.001
7. Although more time would be required to do so, I would feel a sense of accomplishment in treating unique patients like Chang	2.51	1.28	2.41	1.21	0.474	2.43	1.24	2.54	1.27	0.498	2.61	1.22	2.05	1.22	< 0.001
8. I would like to conduct further research on unique patients like Chang	2.53	1.20	2.33	1.15	0.149	2.41	1.15	2.49	1.26	0.605	2.57	1.10	2.07	1.28	0.001

^a^ Scores were reversed such that a higher score indicated a more positive attitude towards individuals with intellectual disability.

^b^ The respondents had experienced caring for people with intellectual disabilities.

^c^ The respondents had family members or friends who are people with intellectual disabilities.

^d^ The respondents had received training on caring for people with intellectual disabilities.

We noted that respondents who had experience in treating or caring for people with intellectual disabilities had significantly lower scores (less favourable attitudes) for the item ‘I would wish Chang were not my patient’ (*p* = 0.021). Having family or friends with intellectual disabilities did not influence the scores assigned to any of the items. Compared with those who did not, respondents who had previously attended or participated in activities or workshops organised by organisations for people with intellectual disabilities had significantly lower scores (less favourable attitudes) for all items, with the exception of the item ‘Patients like Chang require special expertise, so I would refer them to other professionals’.

Table 5 presents the predicted scores estimated for the three groups by using linear regression. Regarding attitudes towards people with intellectual disabilities in nonclinical settings, the predicted scores did not differ significantly between the three groups. Regarding attitudes towards the hypothetical service users with intellectual disabilities in the clinical setting, the ophthalmologists had a significantly higher score than did the other two groups (mean = 23.5; SD = 0.86).

**TABLE 5:** Predicted score by occupation type.^a^

	Predicted score	SD	95% Confidence interval		*p*
General attitude score					
Ophthalmologist	55.87	1.50	52.91	58.83	< 0.001
Optometrist/optician	57.21	0.93	55.38	59.03	< 0.001
Others	56.56	0.91	54.77	58.36	< 0.001
Clinical setting attitude score					
Ophthalmologist	23.48	0.86	21.95	25.02	< 0.001
Optometrist/optician	19.71	0.93	18.06	21.37	< 0.001
Others	17.80	0.86	16.27	19.34	< 0.001

^a^ The scores were estimated using the linear regression model.

## 4 Discussion

This study compared ophthalmologists' general attitudes towards people with intellectual disabilities in nonclinical settings and their attitudes towards these people in a clinical setting. Our key findings are as follows: (1) The ophthalmologists had positive general attitudes towards people with intellectual disabilities in nonclinical settings. (2) Compared with optometrists/opticians and other health‐care professionals, the ophthalmologists had more positive attitudes towards people with intellectual disabilities in the clinical setting. (3) Nevertheless, the ophthalmologists had negative attitudes towards the people with intellectual disabilities in the clinical setting, as reflected by their responses to some of the questionnaire items. The preceding findings support our hypotheses H1 and H2. (4) Having experience in taking care of people with intellectual disabilities or having previously participated in activities or workshops organised by organisations for people with intellectual disabilities was associated with unfavourable attitudes towards people with intellectual disabilities. This finding does not support our hypothesis (H3).

This study has several strengths. First, this study provides empirical evidence regarding ophthalmologists' attitudes towards people with intellectual disabilities; this topic has rarely been investigated in the literature. Second, we included a comparative group, which enabled us to compare the attitudes of ophthalmologists with those of other health‐care professionals. Third, we collected data on both attitudes in nonclinical settings and those in a clinical setting; our findings provide valuable information that can guide policy formulation. Strategies for improving attitudes in everyday life may differ considerably from strategies aimed at improving clinical care for people with intellectual disabilities. The nuanced understanding of such attitudes presented in this study can guide the development of targeted policy interventions.

Reluctance to treat people with intellectual disabilities does not necessarily indicate discrimination. Ophthalmologists, as with other physicians, must exercise discretion in their treatment decisions, and their on‐the‐spot clinical decisions are frequently justifiable (Lagu et al. 2022). Our findings contribute to this understanding by revealing that the participants in all three occupational groups generally held favourable attitudes towards people with intellectual disabilities in nonclinical settings. This underscores our argument that a critical distinction exists between general attitudes and those exhibited in a clinical setting.

No previous study has investigated whether attitudes towards people with intellectual disabilities are influenced by physicians' experience in treating or caring for people with intellectual disabilities or their participation in activities or workshops held by organisations for people with intellectual disabilities. Understanding these influences is crucial because individuals often adjust their performance expectations on the basis of the actual tasks they anticipate being required to perform (Armor and Sackett 2006).

Studies on physicians' and health‐care professionals' attitudes towards people with intellectual disabilities have reported inconsistent findings. Some studies have revealed that physicians generally have negative attitudes towards people with intellectual disabilities (Bacherini et al. 2021), whereas others have noted that physicians exhibit positive attitudes (Morin et al. 2018). The present study contributes to the literature by revealing that health‐care professionals exhibited significantly negative attitudes towards people with intellectual disabilities in clinical settings compared with nonclinical settings. One possible reason is the reimbursement structure of Taiwan's National Health Insurance programme; in general, the compensation health‐care professionals receive under this programme does not adequately reflect the time, effort, equipment costs, and necessity of a multidisciplinary team required for treating a service user with intellectual disabilities. This might explain the reluctance among many participants towards treating the hypothetical service user with intellectual disabilities. Reimbursement affects financial incentives and therefore physician practice (Shrank et al. 2005).

In ophthalmology, reimbursement systems that do not account for time and costs associated with complex cases might inadvertently encourage a preference for simpler cases (Portney et al. 2023), such as those involving people without disabilities. Therefore, to cultivate a more accommodating clinical environment for people with intellectual disabilities, the reimbursement rates for the treatment of these people should be increased. One approach to adjusting these rates could be the establishment of specialised clinics that qualify for superior compensation rates. Taiwan's Ministry of Health and Welfare established a pilot eye care clinic in a medical centre in northern Taiwan for the treatment of vision problems in children with special needs, including those with intellectual disabilities (Wu et al. 2022). More of these clinics should be established to improve access to eye care for people with intellectual disabilities. In addition, only a specific group of ophthalmologists would require training for providing these specialised services in these clinics, rather than all ophthalmologists, which may not be feasible under Taiwan's current National Health Insurance programme.

Physicians, particularly those in Taiwan, often face considerable time constraints in their practice (Tai‐Seale et al. 2007). The Taiwanese health‐care system is characterised by a high volume of outpatient visits and short consultation durations per outpatient episode (Wu et al. 2010), and this makes the treatment of people with intellectual disabilities, who often require extended consultation time, challenging and thus undesirable. Furthermore, patients should be cooperative during ophthalmological examinations and treatments. However, in standard medical education, ophthalmologists do not receive training on understanding people with intellectual disabilities and learning how to communicate with these people; thus, ophthalmologists are required to make extra efforts to learn how to communicate with these people. Specialised equipment or devices, such as portable slit lamps, portable refractometers, retinoscopes, symbol acuity charts, or preferential looking acuity charts, are often required when providing care to people with intellectual disabilities. Ophthalmologists are required to put in extra time and effort to obtain this information.

A study revealed that health professionals reported a lack of confidence in providing care for people with intellectual disabilities, with this lack of confidence often stemming from a lack of familiarity with and knowledge about people with intellectual disabilities (Pelleboer‐Gunnink et al. 2017). This is consistent with our finding that among the three occupational groups, the ophthalmologists were more likely to indicate that they would be required to refer the hypothetical patients to other professionals.

We discovered that participants who reported attending or participating in activities or workshops had significantly less favourable attitudes towards people with intellectual disabilities. One potential reason for this is that such activities or workshops might heighten participants' awareness of the specialised care required for people with intellectual disabilities, emphasising the challenges associated with their treatment. This heightened awareness could subsequently make them more hesitant to engage with people with intellectual disabilities. Nevertheless, specialised training focused on enhancing clinical skills for treating people with intellectual disabilities is crucial. A study revealed that in specific contexts, training interventions can effectively improve attitudes towards people with disabilities among health‐care professionals (Ryan and Scior 2014). We suggest that the content of such training should emphasise positive aspects—such as strategies that can assist physicians in accommodating people with intellectual disabilities—in addition to acknowledging inherent challenges, including the additional effort required for treatment of people with intellectual disabilities.

Notably, healthcare professionals' attitudes towards treating people with intellectual disabilities may be influenced by institutional‐level factors, including environments that are not disability‐friendly. These factors might be beyond a healthcare professional's control. Therefore, although training for healthcare professionals is vital, it is not the sole solution to improving their attitudes towards treating people with intellectual disabilities.

This study has some limitations that should be considered. First, we did not employ probability sampling, and therefore, our findings might not be generalisable across the entire country. We were unable to determine the response rate because we recruited participants through convenience sampling. Second, our sample size was determined primarily on the basis of the research budget. The findings deemed statistically nonsignificant in this study might differ in future research with a larger sample. Third, because we recruited participants from professional societies and associated conferences, they were likely to be active members of these societies. This might have introduced social desirability bias given that participants may have responded more favourably to the items related to attitude. Additionally, selection bias may have been introduced because professionals with more positive attitudes towards people with intellectual disabilities are more likely to take the survey. Nevertheless, we observed generally unfavourable attitudes towards people with intellectual disabilities in the clinical setting. Additionally, the use of an extreme example of an individual with intellectual disabilities, as well as ordering response options from most negative to most positive, may have influenced participants' reactions by provoking fear or avoidance responses. Fourth, for practicability, the questionnaires were distributed in paper form and through an online survey via email. This may have introduced bias due to differences in accessibility, response behaviour, and respondent demographics. Finally, the internal reliability of both the clinical and nonclinical scales has been tested, with Cronbach's α values of > 0.8, and their content and face validity have been verified for people with intellectual disabilities. However, the attitude scale used was developed specifically for this study and has not been formally validated.

Perpetuating unequal health care for people with disabilities conflicts with the foundational principles of medicine and public health (Lagu et al. 2022). Ophthalmologists' attitudes towards people with intellectual disabilities can affect vision health inequality among such individuals. Scholars must thus investigate the reasons underpinning such attitudes. Although ophthalmologists may express favourable attitudes towards people with intellectual disabilities in general contexts, this sentiment might not hold in clinical settings. Training for ophthalmologists and other health‐care professionals is vital to ensure they acquire the knowledge and techniques they require to effectively support people with intellectual disabilities. However, the attitudes of ophthalmologists towards people with intellectual disabilities could also be shaped by systemic factors, such as reimbursement protocols. To achieve vision health equity for people with intellectual disabilities, a multifaceted approach must be employed in policy design.

## Ethics Statement

This study was approved by the Institutional Review Board of National Taiwan University Hospital. Approval number: 202306086RINA.

## Conflicts of Interest

The authors declare no conflicts of interest.

## Data Availability

The data that support the findings of this study are available on request from the corresponding author. The data are not publicly available due to privacy or ethical restrictions.

## Appendix A

1. A person says, ‘The best care for people with intellectual disabilities is to be part of normal life in a community’. What do you think of the opinion?
2. A person says, ‘People with intellectual disabilities pose a threat to society because they can pass on their genes for low intelligence’. What do you think of the opinion?
3. A person says, ‘People with intellectual disabilities can have close personal relationships just like everyone else’. What do you think of the opinion?
4. A person says, ‘Since the partner of an “endan” has a family member with intellectual disability, she/he rejects all meetings thereafter expected’. What do you think of the opinion?
5. A person says, ‘The reality of lives of people with intellectual disabilities should be made more widely known to the public’. What do you think of the opinion?
6. A person says, ‘A facility for people with intellectual disabilities should be kept out of a residential area’. What do you think of the opinion?
7. A person says, ‘I do not mind any increase in tax, if it will be used for social services for people with intellectual disabilities’. What do you think of the opinion?
8. A person says, ‘I would not want to live next door to people with intellectual disabilities in the same apartment building’. What do you think of the opinion?
9. A person says, ‘I am willing for my child to have children with intellectual disabilities as close personal friends’. What do you think of the opinion?
10. A person says, ‘I would rather not work with people with intellectual disabilities’. What do you think of the opinion?
11. A person says, ‘Although recently TV programs or newspapers have some topics concerning people with intellectual disabilities, I am not interested because I have no relationship with such people’. What do you think of the opinion?
12. A person says, ‘I would prefer my child not to be made to sit next to a child with intellectual disabilities in a school room because of a possible disturbance for the studies of my child’. What do you think of the opinion?
13. A person says, ‘People without intellectual disabilities should get together more frequently with people with intellectual disabilities’. What do you think of the opinion?
14. A person says, ‘I have a desire to avoid getting involved with persons with intellectual disabilities although you have my sympathy for them and their family’. What do you think of the opinion?
